# *Aspergillus latus*: A cryptic causative agent of aspergillosis emerging in Japan

**DOI:** 10.1093/mmy/myaf052

**Published:** 2025-06-10

**Authors:** Saho Shibata, Momotaka Uchida, Sayaka Ban, Katsuhiko Kamei, Akira Watanabe, Takashi Yaguchi, Vit Hubka, Hiroki Takahashi

**Affiliations:** Medical Mycology Research Center, Chiba University, 1-8-1 Inohana, Chuo-ku, Chiba 260-8673, Japan; Medical Mycology Research Center, Chiba University, 1-8-1 Inohana, Chuo-ku, Chiba 260-8673, Japan; Medical Mycology Research Center, Chiba University, 1-8-1 Inohana, Chuo-ku, Chiba 260-8673, Japan; Medical Mycology Research Center, Chiba University, 1-8-1 Inohana, Chuo-ku, Chiba 260-8673, Japan; Medical Mycology Research Center, Chiba University, 1-8-1 Inohana, Chuo-ku, Chiba 260-8673, Japan; Medical Mycology Research Center, Chiba University, 1-8-1 Inohana, Chuo-ku, Chiba 260-8673, Japan; Laboratory of Fungal Genetics and Metabolism, Institute of Microbiology, Czech Academy of Sciences, Vídeňská 1083, 142 00 Prague 4, Czech Republic; Department of Botany, Faculty of Science, Charles University, Benátská 2, 128 00 Prague 2, Czech Republic; Medical Mycology Research Center, Chiba University, 1-8-1 Inohana, Chuo-ku, Chiba 260-8673, Japan; Faculty of Science, Chiba University, 1-33 Yayoi-cho, Inage-ku, Chiba 263-8522, Japan; Molecular Chirality Research Center, Chiba University, 1-33 Yayoi-cho, Inage-ku, Chiba 263-8522, Japan; Plant Molecular Science Center, Chiba University, 1-8-1 Inohana, Chuo-ku, Chiba 260-8675, Japan

**Keywords:** *Aspergillus latus*, *Aspergillus spinulosporus*, *Aspergillus sublatus*, section *Nidulantes*, aspergillosis

## Abstract

Allodiploid hybrid species, *Aspergillus latus*, belonging to section *Nidulantes*, is a hybrid of *A. spinulosporus* and an unknown species closely related to *A. quadrilineatus* and *A. sublatus*. This hybrid has often been misidentified as the species in section *Nidulantes*, such as *A. nidulans, A. spinulosporus, A. sublatus*, or other cryptic species*. Aspergillus latus* has not been reported in Japan as well as Asia so far. In this study, we screened 23 clinical strains identified as *A. spinulosporus* isolated in Japan from 2012 to 2023 and found seven *A. latus* strains. To characterize the *A. latus* strains, we conducted comprehensive phenotyping including morphological observation, whole genome sequences, and phylogenetic analysis based on calmodulin (*CaM*) gene. In addition, we conducted antifungal susceptibility testing for *A. latus* strains. As a result, the morphological characters of *A. latus* were more similar to those of *A. spinulosporus* compared to *A. sublatus*. However, the ascospore of *A. latus* differed from that of *A. spinulosporus*. Phylogenetic analysis revealed that different *CaM* alleles from the same isolate clustered separately with *A. spinulosporus* and *A. sublatus*, consistent with its hybrid origin. Furthermore, *A. latus* strains showed reduced susceptibility to caspofungin and amphotericin B compared to *A. spinulosporus*, while they were susceptible to azoles. Our results suggest that *A. latus* has been a causative pathogen of aspergillosis in Japan since 2013.

## Introduction

*Aspergillus* species are the causal agents for aspergillosis, mainly affecting immunocompromised individuals.^1^, ^2^ Although *Aspergillus fumigatus* is a main causative agent, other species such as *A. nidulans, A. flavus, A. niger*, and *A. terreus* also contribute to the disease.^3–6^*Aspergillus nidulans*, a member of the section *Nidulantes* poses a serious threat to immunocompromised patients, especially those with chronic granulomatous disease.^3^ Additionally, eleven other species within the section *Nidulantes* have been isolated from patients with invasive aspergillosis.^7^ Recently, Steenwyk et al.^8^, through screening of *A. nidulans* strains, identified that *A. latus* is an allodiploid hybrid species between *A. spinulosporus* and an unknown species closely related to *A. quadrilineatus*. Allodiploid hybrids resulting from interspecific breeding retain the complete genomes of both parental species, potentially contributing to their unique traits. *Aspergillus latus* exhibits higher virulence and minimum inhibitory concentrations (MICs) to some antifungals compared to *A. nidulans*.^8^ This highlights the importance of accurate species identification to ensure effective treatment, as misidentification could compromise patient care.

Steenwyk et al. ^8^, ^9^ reported 30 *A. latus* strains from Belgium, Brazil, France, Germany, Netherlands, and Portugal identified as *A. nidulans, A. spinulosporus*, or other cryptic species, suggesting that *A. latus* infections are likely underreported.^9^ Prospective survey in single center in Japan reported that six *A. nidulans* strains were identified among 107 *Aspergillus* strains.^10^ To date, *A. latus* has not been reported in Asia including Japan. However, *A. nidulans* and some cryptic species were isolated from patients in Japan, namely *A. latus* could have also been isolated previously.^10^, ^11^

In this study, we identified seven clinical *A. latus* strains isolated in Japan by screening 23 *A. spinulosporus* strains which were collected between 2012 and 2023. To characterize these seven *A. latus* strains isolated in Japan, we performed comprehensive phenotyping including morphological observations, whole genome sequences, and phylogenetic analysis based on calmodulin (*CaM*) gene. In addition, we measured the antifungal susceptibility of these strains to six antifungals. Here, we present the first report of *A. latus* being isolated in Japan. Our findings indicate that *A. latus* is a causative agent of aspergillosis in Japan since 2013.

## Material and methods

### Strains

*Aspergillus latus, A. sublatus*, and *A. spinulosporus* strains used in this study have been stored and maintained at the Medical Mycology Research Center, Chiba University (IFM strains) in Japan. Twenty-three clinical Japanese isolates of *A. latus* and *A. spinulosporus* were tested altogether with the type strain of *A. sublatus* IFM 42029 (= IFM 4553 = CBS 140630) collected from soil in Brazil.

### Genomic DNA extraction

Fungi were incubated on Potato Dextrose Agar (Thermo Fisher Scientific, MA, USA) at 30°C for 7 days to obtain fully mature conidia. The conidia were inoculated in Potato Dextrose Broth (Thermo Fisher Scientific) at 37°C at 180 rpm for 16 h. Mycelia were washed with H_2_O, and genomic DNA was purified with phenol-chloroform extraction and Monarch^®^ Genomic DNA Purification Kit (New England Biolabs, MA, USA).^12^, ^13^ The DNA concentration and quality were then measured by Qubit Fluorometer (Thermo Fisher Scientific). The extracted DNA was used for cloning of *CaM* gene and whole-genome sequencing.

### Whole genome sequencing and assembly

Whole-genome 150 bp paired-end sequencing of four *A. latus* strains, IFM 65030, IFM 65233, IFM 65239, and IFM 66778 was performed using Illumina NovaSeq 6000 (Illumina, CA, USA) by Novogene Biotech Co. (Beijing, China). The raw genomic reads were trimmed using fastp v.0.20.1.^14^ The mitochondrial genomes were assembled using GetOrganelle v.1.6.4.^15^ To filter the mitochondrial reads, the reads were aligned with the mitochondrial genome using BWA-MEM v.0.7.17-r1188.^16^ The mapped reads were filtered using SAMtools v.1.10^17^ and SeqKit v.0.10.1.^18^ The nuclear genomes were assembled using VelvetOptimiser v.2.2.6.^19^

The completeness of draft genomes was evaluated by BUSCO v. 5.2.1^20^ with the database eurotiales_odb10. blastn v.2.15.0 +^21^ was used for extracting the sequences of *CaM* of *A. latus* strains by serving the sequence of *A. nidulans* FGSC A4 as query sequence. The genome sizes of *A. latus* strains were estimated using Jellyfish^22^ and GenomeScope with 21 k-mers.^23^ Gene prediction for *fks1* was performed by using WebAUGUSTUS with the model aspergillus_nidulans.^24^

### DNA cloning and sequencing

Two *CaM* genes from three strains (IFM 61956, IFM 63852, and IFM 64360) were PCR-amplified using CF1L_SacI (5′-AAAGAGCTCGCCGACTCTTTGACYGARGAR-3′) and CF4_SacI (5′-AAAGAGCTCTTTYTGCATCATRAGYTGGAC-3′) primers,^25^, ^26^ which contain SacI restriction site. The PCR products were purified and cloned into the pMK-dGFP vector,^27^ followed by transformation into *Escherichia coli* DH5α. Plasmid DNA was extracted and used as a template for Sanger sequencing with PtrpC-Rev (5′-AAATGCTCCTTCAATATCATCTTCTGTCGA-3′) and M13R (5′-CAGGAAACAGCTATGAC-3′) primers.^28^

### Phylogenetic analysis

The sequences of *CaM* ranging from 438 to 824 bp in length were aligned with MAFFT v.7.508.^29^, ^30^ Aligned sequences were trimmed using trimAl v.1.4.rev15,^31^ resulting in 686 bp in length. The maximum likelihood tree of *CaM* was constructed using the multithreaded version of RAxML v.8.2.12,^32^ the GTRCAT model, and 1000 bootstrap replicates. The phylogenetic tree was visualized using the FigTree (free download available at http://tree.bio.ed.ac.uk/software/figtree/). A total of 112 taxa were included in our tree (Supplementary Table 1).

### Morphological observations

*Aspergillus latus* IFM 65329 was used for microscopic and macroscopic observation. The colony character of the other six strains of *A. latus, A. sublatus* IFM 42029^T^ (= IFM 4553^T^), and *A. spinulosporus* IFM 66771 were observed. The observations of colony morphologies were curried on the agar media, i.e., Czapek yeast autolysate agar (CYA, Czapeck Dox Agar, Duchefa Biochemie, Haarlem, Netherlands and Difco™ Yeast Extract, Thermo Fisher Scientific), oatmeal agar (OA, Sigma Aldrich, MO, USA) and malt extract agar (MEA, Thermo Fisher Scientific) with trace elements (0.1 g ZnSO_4_·7H_2_O and 0.5 g CuSO_4_·5H_2_O in 100 ml distilled water) at 25°C or 37°C for 7 days. Microscopic observations of conidia and conidiophores were made from 1-week-old colonies on MEA. Ascomata and ascospores were observed from 8-week-old colonies on OA. A light microscope (Nikon ECLIPSE Ni, Tokyo, Japan and Swift Optical Instruments S7-TP520, TX, USA) was used to examine morphological characters including the size and shape of ascomata, ascospores, conidiophores, and conidia. These were mounted in a drop of water on glass slides. The slide preparations were examined and photographed using a digital camera (Nikon DS-Fi3, Japan). Approximately 30 structures were randomly chosen, and their length and width were measured using ‘ImageJ’ software (free download available at http://rsbweb.nih.gov/ij/).

### Antifungal susceptibility testing

MIC tests for amphotericin B (AMPH-B), itraconazole (ITCZ), voriconazole (VRCZ), micafungin (MCFG), and caspofungin (CPFG), were performed using the Dried Plate for Antifungal Susceptibility Testing of Yeasts (Eiken Chemicals, Tokyo, Japan). The method follows the Clinical and Laboratory Standards Institute M38-Ed3 with slight modifications.^33–35^

## Results

### Screening and identification of *Aspergillus latus*

To identify the presence of allodiploid hybrid strains of *A. latus*, we sequenced the *CaM* genes of 23 *A. spinulosporus* strains. Sequences with double peaks were observed in seven strains. The sequences of two copies of *CaM* were determined in three strains by cloning or in four strains by whole-genome sequencing (Table 1). These strains were isolated from the patients between 2013 and 2022. The ages of the patients ranged from 13 to 79 years. The patients were diagnosed with invasive pulmonary aspergillosis (IFM 63852 and IFM 66778) and chronic pulmonary aspergillosis (IFM 65233 and IFM 65329), the other patients were not diagnosed with aspergillosis (IFM 61956, IFM 64360, and IFM 65030). However, the patients were diagnosed with other diseases such as leukemia. Therefore, we could not conclude that *A. latus* is the primary cause of death.

**Table 1.** Clinical information of *Aspergillus latus* strains examined in this study.

	Species name						
Strain no.	This study	Previously identified	Isolation year	Source	Disease record	Age	Outcome
IFM 61956	*A. latus*	*A. spinulosporus*	2013	sputum	colon cancer, pneumonia	N/A	dead
IFM 63852	*A. latus*	*A. spinulosporus*	2016	sputum	interstitial lung disease, invasive pulmonary aspergillosis	79	dead
IFM 64360	*A. latus*	*A. spinulosporus*	2016	BALF	ulcerative colitis, pneumonia	45	dead
IFM 65030	*A. latus*	*A. spinulosporus*	2017	sputum	bronchiolitis	62	alive
IFM 65233	*A. latus*	*A. spinulosporus*	2018	lung	liver cirrhosis, chronic pulmonary aspergillosis	66	alive
IFM 65329	*A. latus*	*A. spinulosporus*	2015	sputum	pulmonary non-tuberculous mycobacteriosis, chronic pulmonary aspergillosis	68	alive
IFM 66778	*A. latus*	*A. spinulosporus*	2020	bronchoscope	acute myeloid leukemia, invasive pulmonary aspergillosis	13	alive

Draft genomes for four *A. latus* strains (IFM 65030, IFM 65233, IFM 65239, and IFM 66778) were generated, with assembled genome sizes ranging from 62.1 to 63.2 Mb, aligning with the typical genome sizes of *A. latus* (previous study showed average genome size 69.09 ± 5.68 Mb^8^) (Supplementary Table 2).

### Phylogenetic analysis

**Figure 1.**
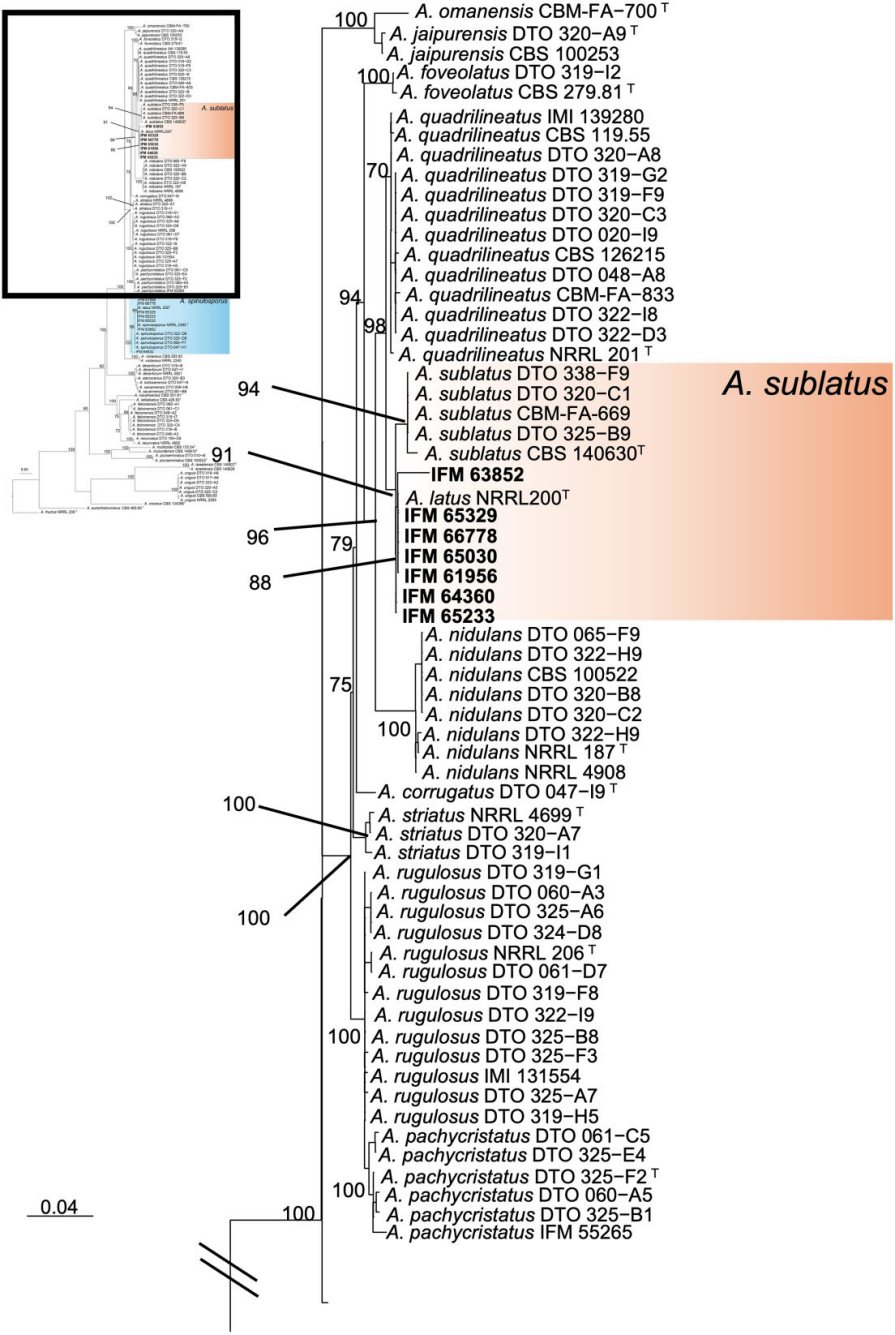

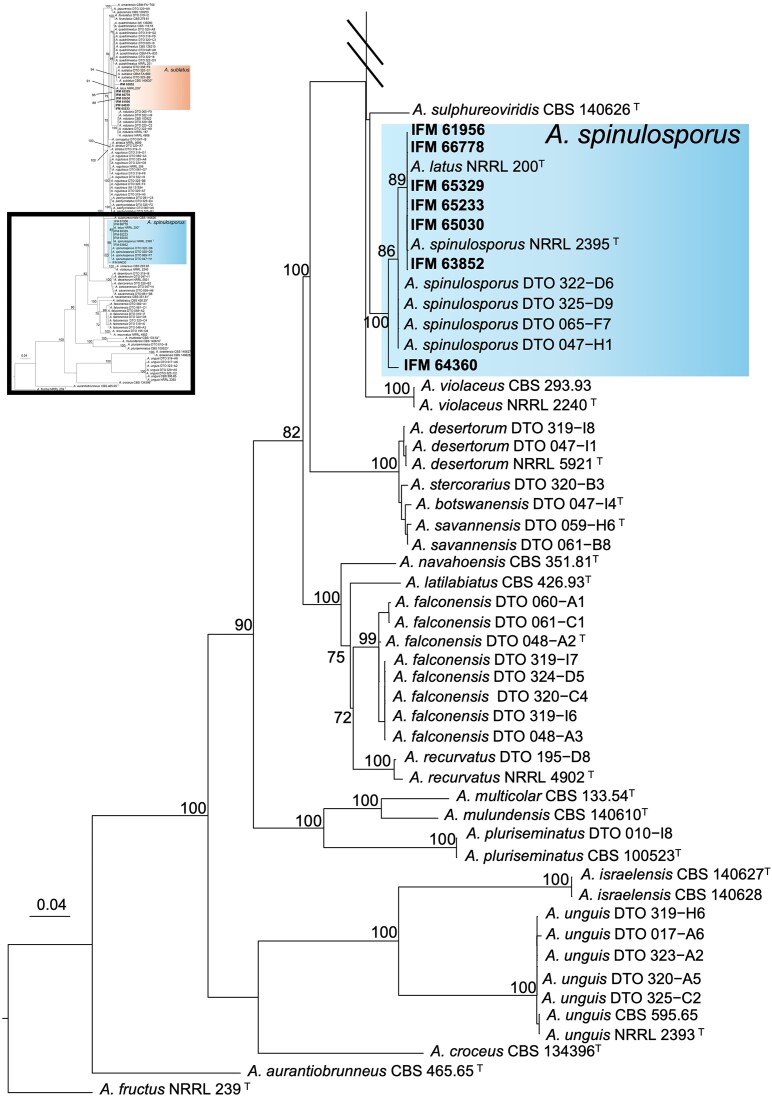
Phylogenetic tree was constructed using maximum likelihood (ML) analysis based on the models selected with the GTRCAT for *CaM* (Bootstrap value: >70%). *Aspergillus aurantiobrunneus* and *A. fructus* were used as outgroups. Numbers after taxa are isolate, specimen, or strain numbers. The scale bar represents the number of nucleotide substitutions per site. The sequences obtained in this study are shown in bold. T = type sequence.

We conducted a phylogenetic analysis to characterize the evolutionary position of seven Japanese *A. latus* strains in relation to closely related species within the section *Nidulantes*, including *A. sublatus, A. quadrilineatus*, and *A. spinulosporus*. Because hybrid species like *A. latus* may carry divergent copies of nuclear genes inherited from different parental species, we cloned the *CaM* gene to isolate and sequence each allele separately. One copy of *CaM* gene sequence clustered with the sequences of *A. spinulosporus*, while the other copy clustered with *A. sublatus* (Fig. 1). The *CaM* sequence of *A. latus* IFM 65329 differed from that of *A. sublatus* CBS 140630^T^ = IFM 42029 (accession no. KU866804) in length by 1 bp and shared 99.12% identity; it differed from that of *A. quadrilineatus* NRRL 201^T^ (accession no. EF652345) in length by 6 bp and shared 98.83% identity. However, the *CaM* sequence of *A. latus* did not contain any species-specific substitution compared to *A. sublatus, A. quadrilineatus*, and *A. spinulosporus*.

### Morphological observations

**Figure 2.**
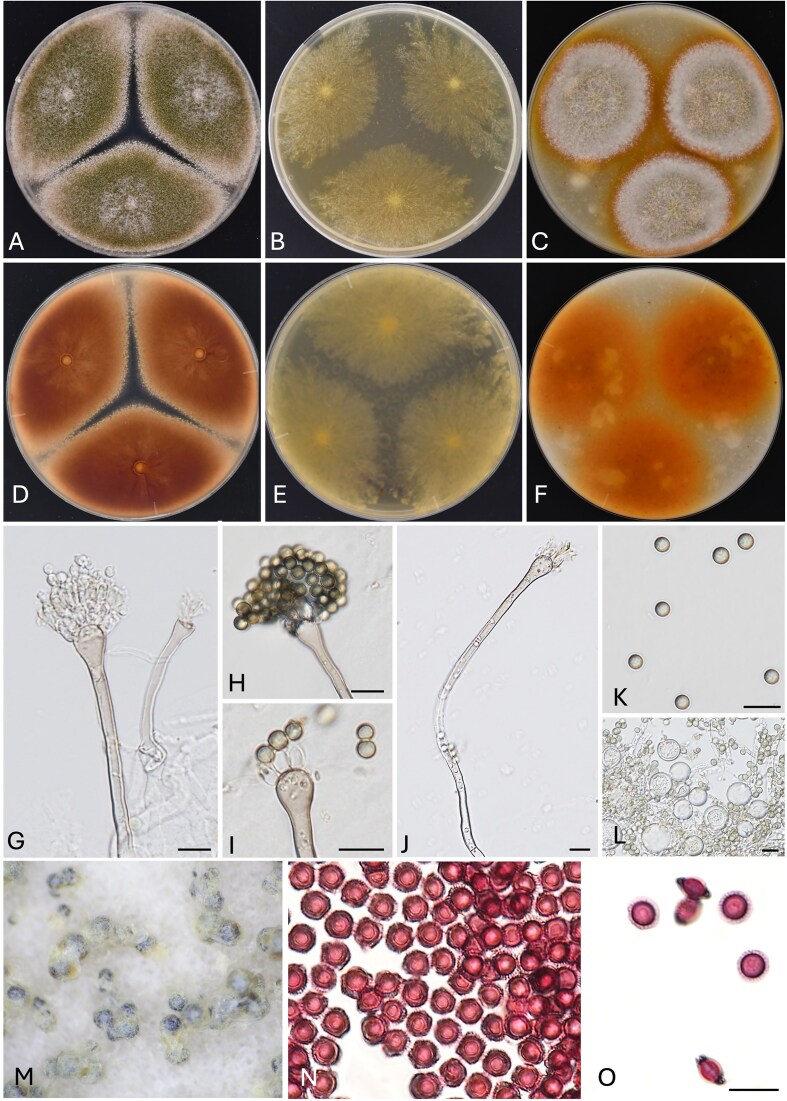
*Aspergillus latus* IFM 65329 colonies: obverse CYA 37°C (A), MEA 37°C (B), OA 25°C (C), reverse: CYA 37°C (D), MEA 37°C (E), OA 25°C, 7d (F), conidiophores (G–J), conidia (K), Hülle cells (L), ascomata (M), and ascospores (N, O). Scale bars: G–L, N, O 10 µm.

The colony morphologies of *A. latus* varied among strains (Fig. 2, Supplementary Fig. 1). On MEA, the growth was slower compared to that of *A. sublatus* and *A. spinulosporus* (Fig. 2B, Supplementary Fig. 1B, E, H, K, N, Q, T, W). In terms of colony appearance, *A. latus* was more similar to *A. spinulosporus* than to *A. sublatus*, as its colonies were covered with green conidia on OA and sometimes on CYA (Table 2, Fig. 2A, C, Supplementary Fig. 1C, D, F, I, L, O, R, X). The microscopic of *A. latus* also closely resembled those of the other two species but ascospores of *A. spinulosporus* are echinulated (Table 2).^36^, ^37^

**Table 2.** Morphological comparison of *Aspergillus latus, A. spinulosporus*, and *A. sublatus*.

		*A. latus* This study	*A. spinulosporus* This study &^36^	*A. sublatus* This study &^37^
Colony	CYA	Obverse: yellow green, olive green, pale brown edge Reverse: dark brown, yellowish brown, green conidia formed in the center	Obverse: pale brown, yellow edge, grey, green conidia forming all over Reverse: pale brow, brown edge	Obverse: reddish brown, grey, pale brown edge, reddish conidia forming in the center Reverse: pale brown, orange, brown edge
	MEA	Obverse: pale yellow, irregular edge with white mycelia Reverse: pale-yellow, soluble yellowish pigments present and exudates absent	Obverse: concentrically growth, pale-yellow Reverse: pale yellow, soluble pigments and exudates absent	Obverse: concentrically growth, pale-yellow, white edge, olive-green conidia formed in the center Reverse: pale yellow, no soluble pigments and exudates
	OA	Obverse: pale brown, green, partially reddish, covered in green conidia, the center pigmented yellow to brown Reverse: soluble orange or brown pigments present and exudates absent	Obverse: pale brown with olive green conidia and sometime wrinkled Reverse: orange to brown, brown soluble pigments present and exudates absent	Obverse: brown with pale brown edge Reverse: orange to brown, brown soluble pigments present and exudates absent
Ascomata	character	cleistothecial, superficial, black, globose, subglobose, surrounded by Hülle cells	cleistothecial, superficial, dark brown, globose, subglobose, surrounded by Hülle cells	cleistothecial, superficial, red to purple, globose, subglobose
Hülle cells	size (μm)	8.7–20 (diameter)	14–30 (diameter)	12.5–30 × 10–28
	character	hyaline, globose to ovoid	hyaline, globose to ovoid	hyaline, globose to ovoid
Ascospores	size (μm)	3.5–10 (pleated equatorial crests: 0.6–2.3)	3.5–4.5 × 3.0–4.5 (pleated equatorial crests: 0.8–1.0)	4.0–4.5 × 3.0–3.5 (pleated equatorial crests: 1.0–1.5)
	character	purple red, reddish brown, in surface view globose, subglobose, spore bodies smooth, incompletely reticulate, ribbed, globose, subglobose, in side view lenticular, with two pleated equatorial crests	orange,reddish brown, in surface view globose, subglobose, spore bodies echinulate, globose, subglobose, in side view lenticular	purple red, reddish brown, globose, subglobose, spore bodies smooth, in side view lenticular
Conidiophores	size (μm)	50–179 × 2.0–3.6	70–120 × 5.0–6.0	200 × 4.0–6.0
	character	smooth stipes, pale brown	smooth stipes, yellowish brown	pale grey, grayish yellow
Vesicles	size (μm)	4.8–9.0 (wide)	9.0–11 (wide)	9.0–12.5 (wide)
	character	pale brown, subglobose to subclavate	yellowish brown, subclavate, fertile over the upper half	hemispherical, flask-shaped
Metulae	size (μm)	5.1–8.3 × 2.0–4.0	6.0–8.0 × 3.0–4.0	4.0–6.0 × 3.0–4.0
	character	hyaline to pale green	pale brown to pale green	hyaline to pale yellowish brown
Phialides	size (μm)	5.2–8.4 × 2.0–4.0	6.0–8.5 × 2.0–3.0	6.0–8.0 × 2.0–3.0
	character	hyaline, flask-shaped	hyaline to pale green, flask-shaped	hyaline to pale yellowish brown
Conidia	size (μm)	2.7–4.8	3.0–4.0	2.5–4.0
	character	echinulate, globose to subglobose, green in mass	echinulate, globose to subglobose, green in mass	echinulate, globose to subglobose, green to pale olive green

### Antifungal susceptibility testing

Antifungal susceptibility testing was conducted on seven *A. latus*, one *A. sublatus*, and three *A. spinulosporus* strains (Table 3). Reduced susceptibility to CPFG was observed in all tested strains, geometric mean MICs of 7.2 for *A. latus*, 16 for *A. sublatus*, and 5.0 for *A. spinulosporus*. Reduced susceptibility to AMPH-B was noted in *A. latus* and *A. sublatus*, while *A. spinulosporus* strains displayed low MICs, with geometric mean MICs of 4.4, 4.0, and 0.7, respectively. *Aspergillus latus* strains displayed low MICs to MCFG, whereas MICs of *A. sublatus*, and *A. spinulosporus* were elevated. Susceptibility to VRCZ varied: reduced susceptibility to VRCZ was observed in *A. latus* IFM 66778 strain (MIC 2.0). All tested strains were susceptible to ITCZ.

**Table 3.** MIC values for antifungal drugs.

		MIC (µg/ml)				
Species name	Strain no.	MCFG	CPFG	AMPH-B	ITCZ	VRCZ
*Aspergillus latus*	IFM 61956	0.06	16	4	0.5	0.12
	IFM 63852	0.12	16	4	0.25	0.12
	IFM 64360	0.06	16	4	0.25	0.25
	IFM 65030	0.06	4	4	0.25	0.06
	IFM 65233	0.06	2	4	0.5	0.06
	IFM 65329	0.03	8	4	0.25	0.12
	IFM 66778	0.06	4	8	0.25	2
*A. sublatus*	IFM 42029^T^	>16	16	4	0.25	0.12
*A. spinulosporus*	IFM 66771	0.15>	2	1	0.25	0.25
	IFM 61449	>16	8	0.5	0.25	0.12
	IFM 64750	>16	8	1	0.25	0.12

MCFG = micafungin, CPFG = caspofungin, AMPH-B = amphotericin B, ITCZ = itraconazole, VRCZ = voriconazole.

## Discussion

Here, we report the allodiploid hybrid *Aspergillus latus* strains isolated in Japan. Although 30 strains have been found worldwide,^8^, ^9^*A. latus* has not been reported in Japan as well as Asia. By screening 23 *A. spinulosporus* strains, we found seven clinical *A. latus* strains (30.4%). *Aspergillus latus* IFM 61956 was isolated in 2013, suggesting that this species might be more prevalent in the country. The pathogenicity of *A. latus* was shown to be comparable to that of *A. spinulosporus* and *A. nidulans*.^8^, ^9^ In this study, the pathogenicity of *A. latus* varied among cases, with some strains identified as colonizers and others associated with defined clinical diseases such as invasive aspergillosis and chronic pulmonary aspergillosis (Table 1). However, ongoing monitoring is necessary, as changes in pathogenicity or resistance profiles could impact treatment strategies in the future.

We conducted a phylogenetic analysis of *A. latus* (Fig. 1) with results indicating that it is derived from a hybridization between *A. spinulosporus* and a species related to *A. sublatus* and *A. quadrilineatus*. Morphological characters of seven *A. latus* strains, such as colony color and growth on MEA, were the most stable among strains (Fig. 2B, Table 2, and Supplementary Fig. 1B, E, H, K, N, Q). The differences between *A. latus, A. spinulosporus*, and *A. sublatus*, however, were slight, and given the variability observed in previous studies, species identification relying on these traits may be challenging (Supplementary Fig. 1).^9^

In terms of antifungal susceptibility, strains of *A. latus* found in Japan demonstrated increased susceptibility to ITCZ compared to overseas strains,^8^ while showing reduced susceptibility to CPFG, aligning with the previous research^8^, ^9^ (Table 3). It has been reported that *A. spinulosporus* exhibits reduced susceptibility to CPFG compared to *A. nidulans* and other clinically relevant species in section *Nidulantes*.^8^, ^38^ In this study, we confirmed that *A. spinulosporus* and *A. sublatus* showed reduced susceptibility to CPFG, suggesting that this characteristic in *A. latus* may be derived from these species. On the other hand, *A. latus* retained high susceptibility to MCFG, another echinocandin antifungal. While point mutations in the hotspot 1 of *fks1* have been implicated echinocandin resistance in *A. fumigatus* and *Candida* species,^39^ no such mutations were detected in *A. latus*. Instead, multiple amino acid changes, including insertions or deletions ranging from 1 to 22 residues, were identified in a different region of Fks1 (Supplementary Fig. 2).^24^ Interestingly, these mutations were present in only one of the two homologous *fks1* alleles in *A. latus*. Further studies are needed to elucidate the potential role of these unique mutations in modulating susceptibility to echinocandin antifungals.

In addition, *A. latus* strains exhibited AMPH-B resistance. Although high AMPH-B MIC values have been reported in other *Aspergillus* species, especially *A. nidulans* and related species,^8^, ^38^, ^40^ the mechanisms underlying the resistance remain poorly understood. Increased catalase production has been implicated in AMPH-B resistance in *A. terreus*.^41^ Whether a similar mechanism operates in *A. latus* remains unknown and warrants further investigation.

In conclusion, our study provides significant insights into the ecology and clinical relevance of *A. latus*, a newly recognized hybrid pathogen and cryptic species of *A. nidulans*. We documented the first occurrence of this pathogen in Japan, demonstrating its prevalence and potential role as a causative agent of aspergillosis since 2013. The comprehensive phenotyping, including morphological and genetic analysis, underscored the diagnostic challenges due to its similarities with *A. spinulosporus* and *A. sublatus*. Moreover, antifungal susceptibility testing revealed reduced susceptibility to certain antifungal agents, highlighting the need for accurate identification to inform effective treatment strategies. To support clinical mycology laboratories, we suggest that when sequencing of multiple genetic loci reveals the presence of overlapping nucleotide signals, i.e., W-type peaks, and BLAST results show high similarity to *A. latus, A. spinulosporus, A. sublatus*, or *A. quadrilineatus*, the possibility of *A. latus* should be considered. The development of reliable molecular diagnostic tools for the accurate identification of *A. latus* will be crucial for effective clinical management. Our findings add to the growing body of knowledge about the distribution and pathogenic characteristics of *A. latus* and emphasize the importance of continued surveillance and accurate diagnostics in clinical mycology.

## Supplementary Material

myaf052_Supplemental_Files
