# Supplementary figures and images for: Aspergillus latus: A cryptic causative agent of aspergillosis emerging in Japan
Source: Med Mycol. 2025 Jun 10;63(6):myaf052. doi: 10.1093/mmy/myaf052 (PMC12188289; doi:10.1093/mmy/myaf052)

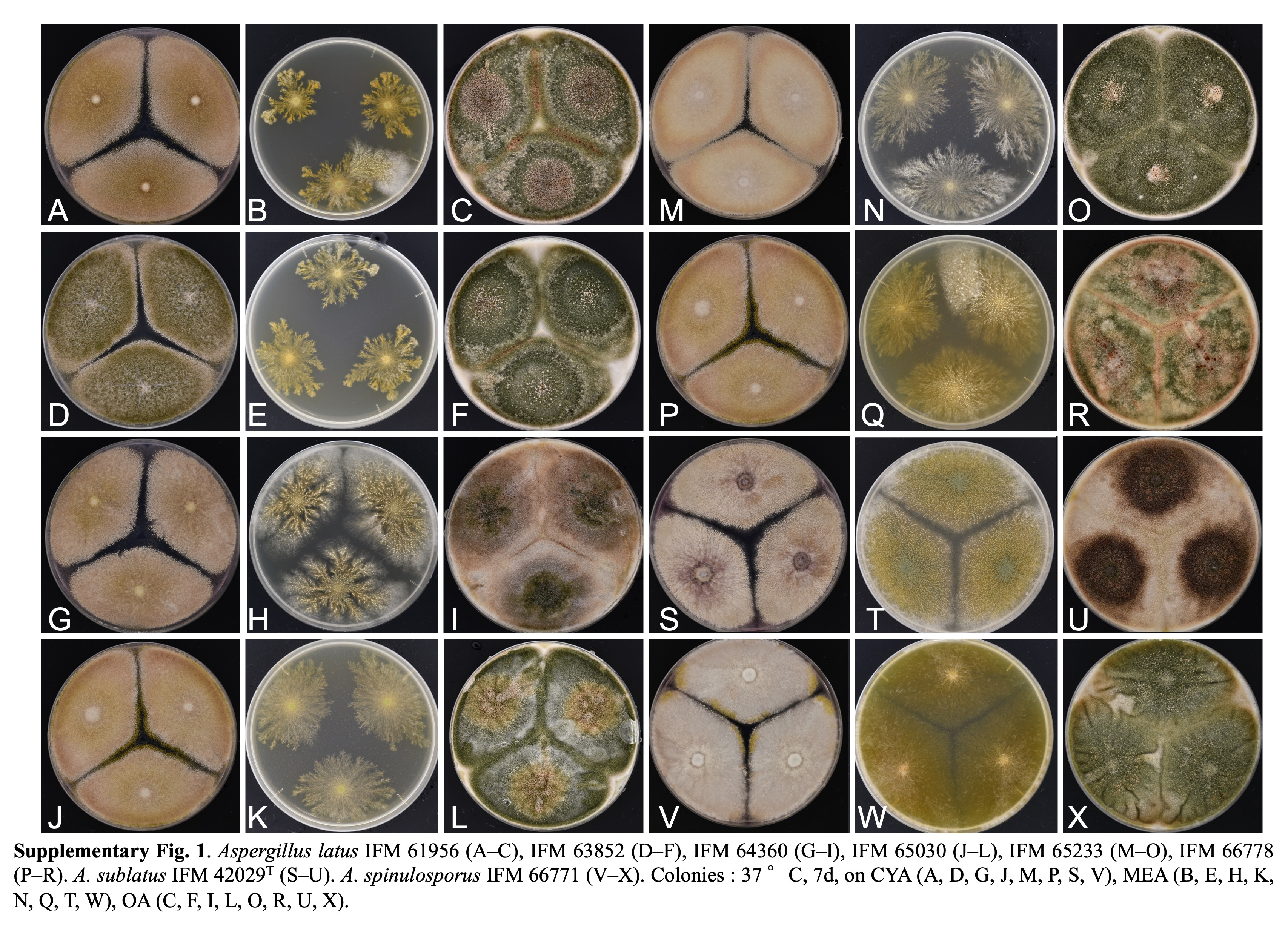

Supplement: myaf052_Supplemental_Files [file myaf052_supplemental_files.zip › mm-2025-0051-File005.tiff]

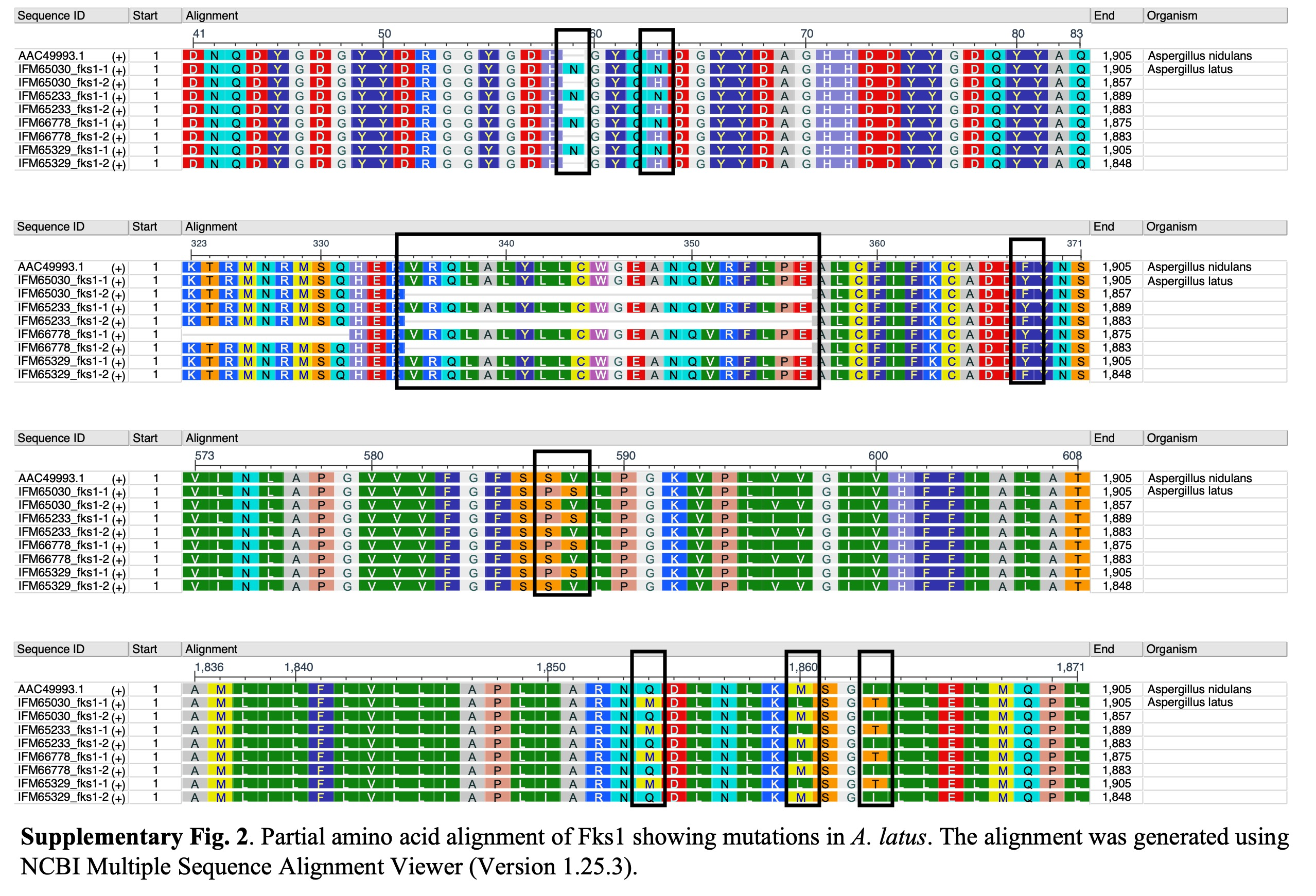

Supplement: myaf052_Supplemental_Files [file myaf052_supplemental_files.zip › mm-2025-0051-File006.tiff]
